# The Role of Polysomnography in Identifying Sleep Disorders in Children with Migraine

**DOI:** 10.25122/jml-2020-0025

**Published:** 2020

**Authors:** Smaranda Antonia Nita, Raluca Ioana Teleanu, Ovidiu Alexandru Bajenaru

**Affiliations:** 1. Clinical Neurosciences Department, “Carol Davila” University of Medicine and Pharmacy, Bucharest, Romania; 2. Neurology Department, University Emergency Hospital, Bucharest, Romania; 3. Pediatric Neurology Department, “Dr. Victor Gomoiu” Children's Hospital, Bucharest, Romania

**Keywords:** migraine, pediatric, polysomnography, REM sleep, N3 - non - REM3, N4 - non - REM4, NREM - non - REM, PSG - polysomnography, REM - Rapid Eye Movement

## Abstract

Migraine pathophysiology and sleep share common neural pathways, and there are clinical as well as paraclinical observations, which lead to the hypothesis of an association between migraine and sleep disorders.

The objective of this study consisted of the evaluation of a possible correlation between migraine and sleep disorders in children, as reflected by sleep architecture and electroencephalographic patterns.

Eighteen patients aged five to seventeen were recruited for the migraine group, and sixteen age-matched patients with no criteria for migraine or any underlying organic disorder, diagnosed with emotional disorders, were enrolled in the control group. All patients underwent inpatient full night polysomnographic recordings, the results of which were analyzed using appropriate statistical methods.

Patients in the migraine group had decreased REM sleep (p = 0.049) and increased N1 sleep (p = 0.018) percentages, compared to the control group. Also, more arousals (p = 0.011) and lower sleep latency (p = 0.029) were noted in the migraine group. A statistically significant association was observed between migraine and sleep disorders when the latter was defined with respect to normal values of polysomnographic parameters published in studies conducted on healthy children.

Polysomnography can be a useful tool for studying sleep in pediatric migraine patients. The results of this study can be regarded as a starting point for a better understanding of the complex role of sleep in the developing brain and of eventual intricacies with migraine pathophysiological mechanisms.

## Introduction

Sleep is a thoroughly investigated subject in neuroscience, for its significant impact on the nervous system function, the extent of which is still under research. The study of sleep in children is challenging when taking into account the practical difficulties but also the complexity of the bidirectional links between sleep and the developing brain.

On the other hand, migraine is a disorder that affects both adults and children, with an impact on their quality of life. Migraine is a rare disorder in children, when it is correctly diagnosed, taking into account the polymorphism of the clinical picture and the thorough differential diagnosis steps which need to be made, in order to rule out other conditions, such as epilepsy, transient ischemic attacks, raised intracranial pressure, posterior fossa tumors, vasculitis, periodic paralysis, acute central nervous system infections, transient metabolic syndromes, ear, nose, throat (ENT) disorders, gastrointestinal disorders and psychiatric disorders.

The relation between sleep disorders and migraine has been a subject for research, mostly in adults, following initial clinical observations, such as sleep deprivation as a trigger for migraine attacks or cessation of migraine attacks after sleep. Also, some studies enquire about the role of sleep deprivation in modulating pain and hyperalgesia [1]. The use of melatonin as a prophylactic drug in migraine has been studied, with similar results, when compared to amitriptyline [2] or valproic acid [3].

The use of polysomnography (PSG) in migraine patients has been studied mainly in adults. Some studies revealed a high non-REM sleep (scored as N3, N4) percentage during nights before morning migraine attacks [4], prolonged sleep latency, a low non-REM (scored as N4) and REM percentage and a high arousal index [5]. One study in children showed less REM and slow-wave sleep in pediatric patients with chronic migraine [6]. Another study, conducted on migraine patients aged under eighteen, revealed disordered sleep due to obstructive sleep apnea, periodic limb movement disorder, central hypersomnia, and sleep architecture disorders, such as higher non-REM 2 (NREM 2) sleep and lower NREM 3 percentages, compared to healthy controls [7]. Other researchers found decreased arousability and low NREM sleep instability in children with migraines without aura [8]. Another reported sleep disorder in children with migraine without aura was periodic limb movement disorder [9].

However, at this moment, there is no consensus on the need to study sleep in patients with migraine, nor on the sleep parameters which should be assessed in order to search for a possible causal relationship or association between migraine and sleep disorders. In children, this task is even more challenging, as sleep has a vital role in their physical, cognitive and behavioral development, and migraine is still a disorder with a complex, multifactorial, and not entirely elucidated pathophysiology and a highly polymorphic clinical picture, especially in children.

The article aims to bring into attention the importance of a complex approach to diagnosing and managing migraine cases, with the use of polysomnography, in the pediatric population.

The objective of this study consisted of the evaluation of a possible correlation between migraine and sleep disorders in children, as reflected by sleep architecture and electroencephalographic patterns.

## Material and Methods

A prospective study was carried out over 28 months (January 1st, 2017 – June 6th, 2019), for which eighteen patients were recruited. The patients were aged five to seventeen and were diagnosed with migraine with aura and migraine without aura, based on the International Classification of Headache Disorders, third edition (beta version) [10]. Patients with respiratory, cardiac, or ENT disorders were excluded.

Because of a paucity of healthy volunteers, the control group consisted of sixteen matched by age patients, with chronic headache syndromes which no migraine criteria, according to the International Classification of Headache Disorders, third edition (beta version). The controls had a normal neurological examination, and they did not have an organic neurologic or systemic underlying disorder, as assessed by clinical examination and full paraclinical work-up, including cerebral magnetic resonance imaging and magnetic resonance angiography) and/or computed tomography. They were diagnosed, with the aid of psychological evaluations, with emotional disorders.

The institutional ethical committee approved the study, and all patients’ caregivers signed written informed consent.

All patients underwent an overnight PSG recording, in inpatient settings, using 26 gold electrodes, placed according to the International 10-20 Electrode Placement System and to the AASM Manual for Scoring of Sleep and Associated Events [11].

The PSG protocol was completed by full scalp electrode placement, in order to have a full account of sleep electroencephalographic patterns, to score sleep stages correctly, as well as to be able to identify possible pathological elements (e.g., epileptiform discharges) which could have pleaded for a more thorough differential diagnosis.

The assessed parameters were: Total Sleep Time (TST), Sleep Latency, REM Latency, Sleep Efficiency, REM, N1, N2, N3 percentages (% TST), Arousals, Wakings After Sleep Onset.

For the analysis and definition of a sleep disorder diagnosis in patients from the migraine group, two parameters were chosen, the values of which were taken from a sizeable polysomnographic study conducted on healthy patients of pediatric age: sleep efficiency < 89% and REM (% TST) < 17 [12].

The results were collected via Microsoft Excel 2010 and statistically analyzed using the Statistical Package for the Social Sciences (SPSS) software (version 21.0). Quantitative numerical variables were expressed as mean +/- standard error of the mean. They were analyzed using the T-Test or One-Way Anova Test. Moreover, the Mann-Whitney (U-Test) and Two-Sample Kolmogorov-Smirnov tests were used to assess groups with a small number of patients.

The distribution of cases was analyzed with Pearson's chi-squared test in order to evaluate the statistical association between binary or multiple logistic data. The Mantel-Haenszel Common Odds Ratio Test (Crosstabs analysis, SPSS) evaluated the odds ratio associated with the distribution in the risk group (patients with migraine), normalized to the control group.

A bivariate parametric correlation was made between different, normally distributed numerical parameters, using the Pearson correlation coefficient and the associated p-value. The Pearson correlation coefficient was interpreted according to the Salkin scale: values between ±0.8-1 – very strong, values between ± 0.6-0.8 - strong, between 0.4-0.6 – moderate, between 0.2-0.4 – low, between 0- 0.2 – no correlation. A p-value of <0.05 was considered statistically significant.

## Results

### General considerations

Migraine cases were more frequent among male patients (68.8%), compared to female patients (38.9%); p = 0.082. There was no statistically significant difference between the age of the patients in the migraine (12 years) versus the control (12.25 years) group, p=0.788.

### Analysis of the main parameters in the migraine group

Migraine with aura was more frequent among female patients (71.4%) versus male patients (18.2%), compared to the mean in the migraine group (38.9%), p = 0.024.

The odds ratio of being diagnosed with migraine with aura was over 11 times higher (1/0.089) if the patient was female, compared to male patients (p = 0.035).

Age was higher among patients diagnosed with migraine with aura (13.57 years), compared to patients with migraine without aura (11 years); p = 0.020.

Migraine with visual aura was more frequent among female patients, compared to male patients, who had, predominately, sensory auras (p = 0.008).

Symptomatology mean duration was higher among migraine with aura patients (22.7 months), compared to patients diagnosed with migraine without aura (15.6 months); p = 0.324.

The frequency of the migraine attacks was similar between patients with migraine with aura and without aura (p = 0.426).

Family history of migraine was present only in patients diagnosed with migraine with aura (p = 0.060).

### Sleep architecture and sleep disorders in patients with migraine

Total sleep time (TST; minutes) was lower in the migraine group, compared to the control group; p = 0.371. TST was lower in the migraine with aura group, compared to the patients with migraine without aura (p = 0.493).

Sleep latency (minutes) was decreased in patients with migraine, compared to the control group; p = 0.029. Sleep latency was lower in patients with migraine with aura (p = 0.742).

REM latency (minutes) was prolonged in patients with migraine, compared to the control group (p=0.565), with no significant difference between patients diagnosed with migraine with aura, versus migraine without aura.

Sleep efficiency (%) was similar between the two groups (migraine versus control group); (p =0.904); however, it was lower in male patients diagnosed with migraine with aura (p = 0.228).

REM (%TST) was decreased in patients with migraine, compared to the control group (p = 0.049). REM (%TST) was lower in patients diagnosed with migraine with aura, compared to patients with migraine without aura (p = 0.212) and it was higher in male patients, compared to female patients diagnosed with migraine without aura (p = 0.035). REM (%TST) was decreased in male patients, compared to female patients diagnosed with migraine with aura (p = 0.032) and it was increased in patients with visual aura, compared to patients with sensory aura (p = 0.032).

N1(%TST) was increased in patients with migraine, compared to the control group (p = 0.018) and was increased in male patients, compared to female patients with migraine (p = 0.023).

N2 (%TST) and N3 (%TST) were similar among patients from the two main groups (p = 0.334; p = 0.506). When the mean values of N1, N2, N3 (%TST) were analyzed in respect to the type of aura (visual versus sensory), the following observations were made: N1 and N3 (%TST) were lower in patients with visual aura (p = 0.264; p = 0.124), while N2 was higher in patients with visual aura (p = 0.387).

Arousals were more frequent in patients with migraine, compared to the control group (p = 0.011). Also, they were more frequent in patients diagnosed with migraine with aura (p = 0.072).

Wakings after sleep onset were more frequent in the migraine group (p = 0.428), predominately among patients with migraine with aura (p= 0.324).

### Migraine and sleep disorder diagnosis

There was a statistically significant association (p = 0.041) between the presence of a sleep disorder and migraine. The odds ratio of having a sleep disorder was 5.6 times higher in the migraine group, compared to the control group (odds ratio = 5.60 ± 0.892, 95% CI 0.974-32.198, p=0.054).

## Discussion

Migraine is a rare disorder in children due to the necessity of a thorough and complete differential diagnosis. At this moment, the association between migraine and sleep disorders is recognized, as reflected by studies in different centers around the world, although especially involving adult patients. However, a systematic approach to correctly assessing sleep disorders and to trace a link between the intricacies of the pathophysiological mechanisms of migraine and disordered sleep, especially in children, is lacking.

The challenges of correctly diagnosing migraine, of excluding comorbidities which could have influenced sleep quality and of obtaining high-quality PSG recordings in children, resulted in a relatively small number of patients, which consists of a significant limitation of the study. However, the analysis of the results was tailored to the small cohorts by using adequate statistical tests.

The PSG protocol was adapted along the course of the study, due to the difficulty of obtaining high-quality recordings in children, most of whom refused a classic full sensor recording or whose quality of sleep suffered from it. Therefore, another limitation of the study consists of the fact that other causes of disordered sleep, such as sleep apnea or hypopnea, periodic limb movement disorder, could not be objectively evaluated or excluded.

The statistically significant results of the present study mainly involve REM and N1 sleep, patients in the migraine group having decreased REM sleep, and increased N1 sleep percentages, with respect to the total sleep time, compared to the control group. Also, more arousals and lower sleep latency were noted in the migraine group. These results are partially consistent with previously reported sleep architecture disorders in children with migraine, as reports are not homogenous, some studies revealing low NREM sleep percentages, while others are pointing to high NREM sleep percentages. Disturbances mainly involve NREM2 and NREM3 sleep, as opposed to the present study, which reveals a high NREM1 percentage. A low REM percentage has been previously reported in one study. Low sleep latency has not been previously reported.

Other results point to the necessity of a differentiated approach to migraine with aura patients, also including the study of the parameters according to the type of aura, as results differed among groups. A statistically significant association was observed between migraine and sleep disorders when the latter was defined with respect to the normal values of polysomnographic parameters published in studies conducted on healthy children.

Polysomnography can be a useful tool for studying sleep in pediatric migraine patients. The study of sleep in this age group reveals interesting results, which can be regarded as a starting point for a better understanding of the complex role of sleep in the developing brain and of eventual intricacies with the pathophysiological mechanisms of migraine, perhaps leading to a better understanding of the latter. Broader studies, involving large numbers of children with migraine and healthy volunteers, in different age groups, need to be carried out, in order to better define sleep abnormalities associated with migraine.

## Conflict of Interest

The authors confirm that there are no conflicts of interest.
